# Toward the eco-friendly cosmetic cleansing assisted by the micro-bubbly jet

**DOI:** 10.1038/s41598-024-58968-x

**Published:** 2024-04-08

**Authors:** Yeeun Kang, Jooyeon Park, Hyungmin Park

**Affiliations:** 1 Department of Mechanical Engineering, Seoul National University, Seoul, 08826 Korea; 2 Institute of Advanced Machines and Design, Seoul National University, Seoul, 08826 Korea

**Keywords:** Microbubble, Cosmetics cleansing, Bubbly jet, Venturi tube, Entrainment, Mechanical engineering, Fluid dynamics

## Abstract

While numerous types of chemical cosmetic cleansers have been presented, those with sensitive skin may still experience some irritation while using them. Moreover, the environmental issue of chemical agents has been documented repeatedly. To address these, we suggest the potential application of a micro-sized bubble-laden water jet to cleanse the cosmetics without (or less) using chemical detergents. We devised a venturi-type nozzle with a mesh and air holes capable of generating massive fine bubbles. By testing with the foundation and lip tint (known to be highly adhesive) coated on the synthetic leather and artificial skin surfaces, we measured that the cleansing performance of the bubbly jet is much better (even without the chemical agent) than the single-phase liquid jet. As a mechanism for enhanced removal, it is understood that the greater kinetic energy of the jet due to the acceleration of the effective liquid–air mixture flow and the direct bubble-cosmetic collisions play essential roles. We believe that the present results will spur the development of environment-friendly cleaning methods.

## Introduction

In our daily lives, in general, various chemical agents (e.g., cleansing foam, oil, and tissue) including surfactants are necessarily used to remove the skin makeup of various kinds. These surfactant-based removal agents, however, have a potential of various skin damages and irritations. For example, the outermost layer of the skin (stratum corneum, SC) interacts with the surfactants in several mechanisms: protein denaturation, selective removal of SC lipids, and disruption of SC lipid organization. They result in (i) the increased permeability through the SC lipid bilayers that are responsible for the epidermal barrier function^1^, and (ii) the SC swelling that causes the corneocytes to lose their natural moisturizing factor^2^. Moreover, it is well known that the surfactants bring a pH imbalance of the skin^3^, causing an impairment of the enzyme synthesis needed for the intercellular matrix lipids production^4^. To mitigate the potential skin irritation, previous approaches have in common suggested reducing the concentration of monomers, increasing the size of micelles, mixing various types of surfactant, adding polymers or hydrophobic substances^5^_._ In the same context, it is not a simple and easy task to find or optimize a cleanser that suits each individual skin^5^. Cleansing based on chemical agents may also lead to environmental pollution. Due to the extensive usage and consumption of such substances, a substantial amount of anionic surfactant is released into the environment, producing severe pollution in the rivers^6^ and sea and providing a source of sludge accumulation in the sewage treatment flows^7^. For example, the aquatic animals may be affected by the surfactants; it was noted that the behaviors of the fish are altered to have irregular movements, muscular spasms, and body torsion, affected by the higher concentration of the surfactants^8^.

As an eco-friendly way of accelerating the detachment (removal) of cosmetics toward the minimal usage of chemical agents, we are interested in the mechanical mechanism utilizing the bubble-induced agitation of gas–liquid two-phase flows. In bubbly flows, the agitation caused by the dispersed phase (bubbles) significantly modulates the continuous-phase (liquid) flow in terms of mass, momentum, and heat transport^9–11^. The flows induced and disturbed by the bubbles, known as bubble-induced drift and agitation, respectively, have been investigated widely because of their academic importance, such as the preferential migration of bubbles^12^, nature of pseudo-turbulence^13^, interfacial momentum transport^14^, and so on. Assisted by these properties, bubbly flows have also shown potential in various practical applications to achieve enhanced mixing^15^, mass and heat transfer^16^, and chemical reactions^17^. Indeed, bubble-assisted cleaning has also been investigated as a promising alternative to other methods requiring chemical agents, energy, or sophisticated devices^18–20^. In addition to the substantially enhanced kinetic energy of the flow even with a small void fraction, bubbles in the liquid have important physical properties beneficial to such applications; e.g., large surface area to volume ratio (high mass transfer rate at the interface), long residence time in the liquid, and strong impulse at the instant of bubble rupture^21^. Obviously, the enlarged interfacial area raises the probability of adhesion of the contaminants to bubble surface. The strong pressure waves and liquid jet flows generated during the bubble breakup apply a high impact to the surface, enhancing the separation of contaminants^22,23^. In this context, previous investigations have reported the efficient elimination of Bovine Serum Albumin (BSA) coatings on the solid surfaces using nanobubbles^24,25^, and the microbubbles were shown to be useful in eliminating the oil or solid particles from metal plates^26,27^. Extending the scope of the previous studies on utilizing the gas–liquid two-phase flows in cleaning, we challenge the problem of removing color cosmetics that are combined with highly viscous glycerin and nano-microparticles by assessing and harnessing the hydrodynamics of microbubble jet flow.

In the present study, toward developing bubble-assisted cleaning methodology, we suggested a small-scaled (hand-held) nozzle device capable of issuing the two-phase bubbly jet, of which the bubble size is ***O***(10^1^–10^2^) μm, without requiring additional energy to generate bubbles. To understand and utilize the underlying mechanism, we evaluated the performance of the cosmetic cleansing of the prototype model by performing systematically comparative experiments with typical cosmetics, foundation, and lip tint, coated on synthetic leather and artificial skin surfaces. Rather than performing the parametric study without fundamental analysis, we try to present the physical understanding of how and why the micro-bubbly jet interacts and removes the cosmetics coated on the surface together. We believe that the encouraging results of the present study, as we will discuss below, will provide a new mechanical cosmetic cleansing approach that is both environmentally friendly and less irritating to the skin.

## Results and discussion

### Hydrodynamic characteristics of the bubbly jet

**Figure 1:**
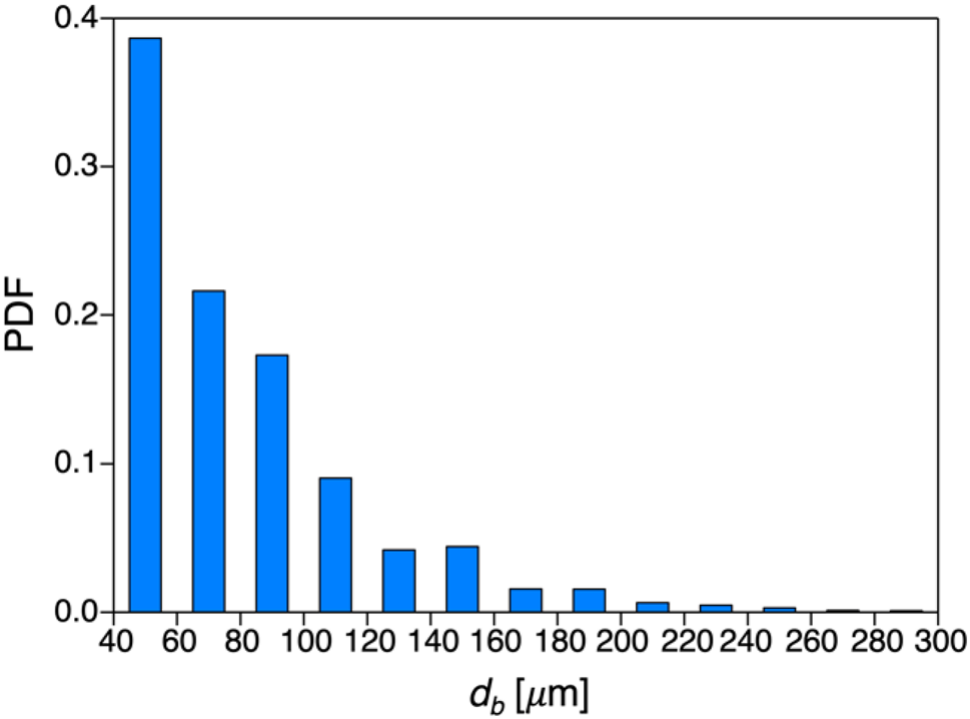
Probability density function (PDF) of the experimentally measured micro-sized bubble diameter inside the bubbly jet.

Before analyzing the capacity of the bubbly jet to remove the cosmetics attached to the surface, we briefly discuss the hydrodynamic characteristics of the present bubbly jet. Figure 1 shows the probability density function of the measured equivalent bubble size, defined as, ${d}_{b}=\sqrt{4{A}_{b}/\pi }$, where *A*_*b*_ is the area occupied by each bubble in the binarized image. As shown, the bubble size spans a wide range from 40 μm to 300 μm (approximately 90% is 40–100 μm), with a mean diameter (${\overline{d} }_{b}$) of 80.7 μm. This is comparable to the bubble size reported for the bubbly jet with similar conditions (water superficial velocity and air flow rate) to the present ones^28^. As it has been reported that microbubbles provide higher cleaning performance compared with millimeter-scaled bubbles^29^, we intended to generate micro-sized bubbles with the present nozzle device. Other properties of the present bubbly jet are summarized in Table 1, which will be discussed in detail below.

**Table 1:** Hydrodynamic properties of the single- and two-phase jets considered in the present study.

	Single-phase liquid jet	Two-phase bubbly jet
Water volume flux (lpm)	4.53 ± 0.12	4.51 ± 0.19
Jet diameter (mm)	12.22 ± 0.68	12.22 ± 0.2
Air volume fraction		
(at the neck)	–	0.125
Time-averaged water jet superficial velocity (m/s)	0.66	> 0.77

Unlike the bubble size, it was not possible to measure the turbulence kinetic energy in the bubbly jet directly, owing to the technical challenges in measuring the water velocity fluctuations of the jet loaded with dense microbubbles (see Fig. 11b, for example). Instead, we tried to estimate the relative change compared to the single-phase jet (without microbubbles) based on the scales of the bubbles and the flow. For bubbly flows with a void fraction of 5–20%, Gore and Crowe^30^ showed that liquid-phase turbulence is attenuated if the bubble size is smaller than 10% of the characteristic length of the flow (e.g., integral length scale). The turbulence reduction by the micro-size bubbles in turbulent flows with the Reynolds number of ***O***(10^3^) has been experimentally confirmed in previous studies as well^31,32^. Considering the size of the present bubble (Fig. 1) and the Reynolds number (~ 9000) of the jet, it is estimated that the velocity fluctuation would be slightly reduced by the bubbles, based on which we further assume that the mechanism of the enhanced cleansing that we will show below is different from the enhanced cleaning performance with macro-scale bubbles (i.e., turbulence augmentation).

### Removal of the foundation

**Figure 2:**
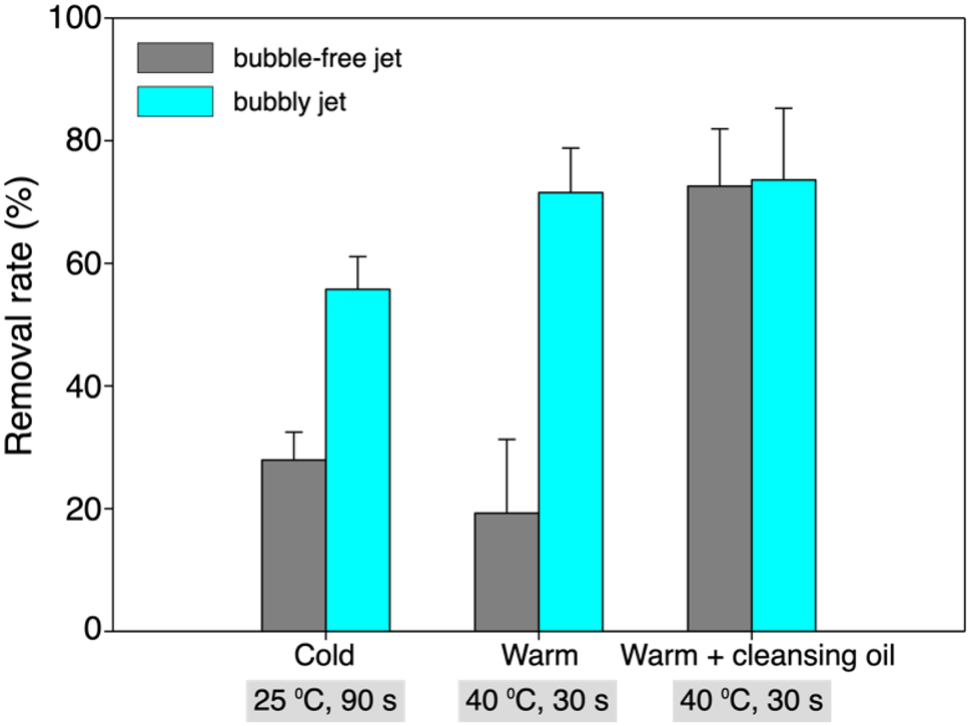
Removal rate of the foundation coated on the synthetic leather depending on the tested conditions (water temperature and duration of the application).

In Fig. 2, we compare the cleaning performance (removal rate) of the foundation coated on the synthetic leather (thickness of 1.2 mm) using the jet with and without bubbles, which was measured for more than five independent experiments. In addition to the existence of bubbles, we also varied the water temperature and time duration for applying the jet. In all cases tested, the cleansing performance of the bubbly two-phase jet is better than that of the single-phase liquid jet. When the water temperature is 25 °C, the removal rate of the foundation by the bubbly jet (56%) is twice that of the liquid jet (28%). As the water temperature increases to 40 °C, interestingly, the difference between the two becomes larger; the removal rate by the bubbly jet increases to 72%, but the liquid jet becomes quite ineffective as it drops to 20%. When the temperature goes high, in general, the properties of the foundation and moisture cream change such that the adhesive energy between cosmetics and the surface is weakened (this is what causes the makeup to come off better in the summer season). Despite the relatively weak adhesion of the foundation, the momentum of the single-phase liquid jet is not strong enough to peel off the foundation from the surface. That is, considering that the cleaning with the warmer water was performed in a shorter duration (30 s) than that with the colder one (90 s), it is reasonable to judge that the performance of the single-phase liquid jet is independent of the water temperature. However, the weakened adhesion is benefited by the bubbly jet as its removal rate increases substantially, indicating that the additional bubble-induced energy in the two-phase jet is strong enough to deal with the cosmetics. With the help of the chemical agent, cleansing oil, the large (removal rate) gap between the two is narrowed down substantially, in which case it is understood that the maximum removal rate has been reached with the tested condition. In other words, the bubbles included in the liquid jet play the role of the chemical cleaning agent; in the case of warmer water condition, the removal rate of the bubbly jet reached 72% without the aid of the cleansing oil, which is comparable to the cases with cleansing oil used.

**Figure 3:**
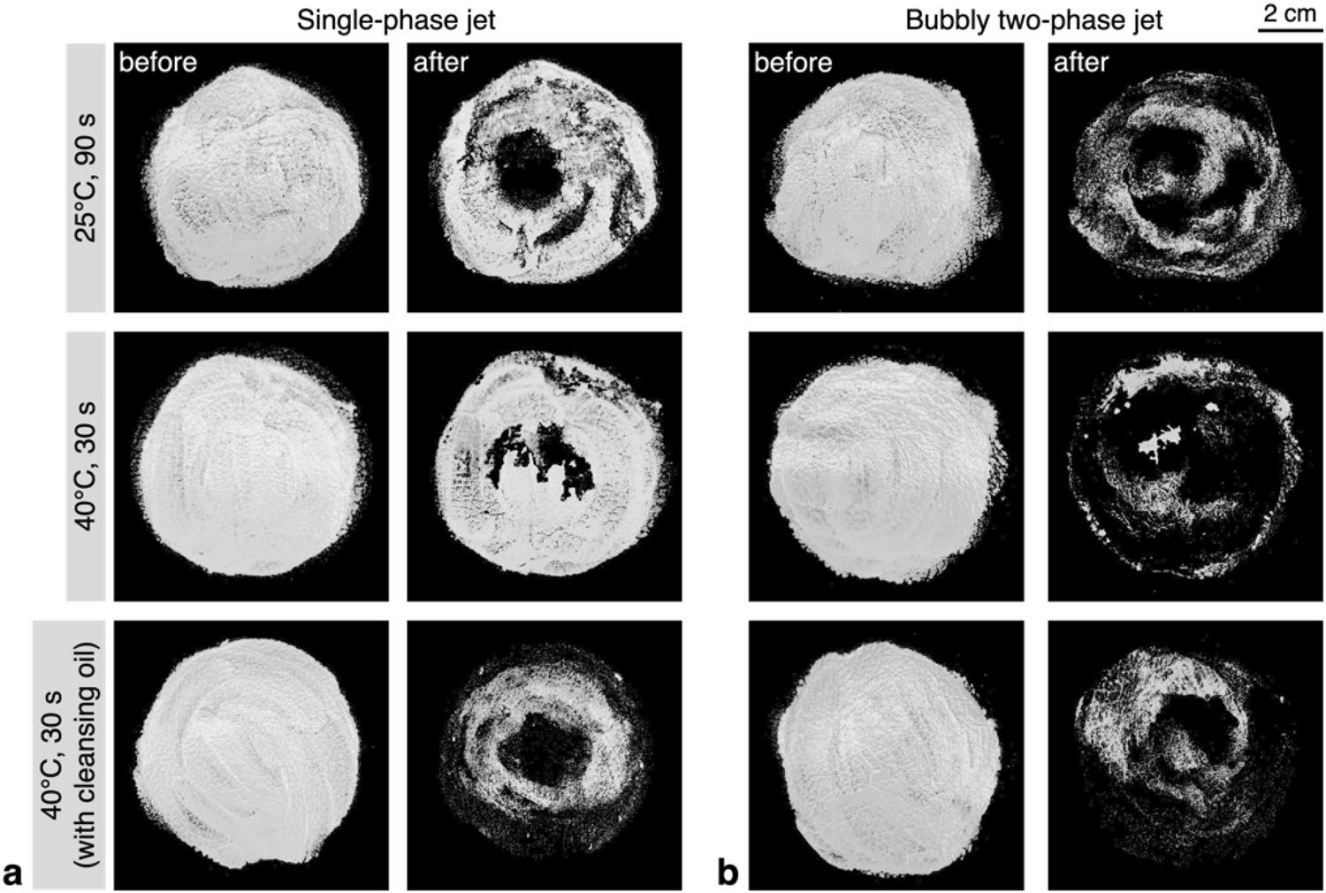
Results of the foundation cleansing test, of which the images were post-processed for quantification: (**a**) single-phase liquid jet; (**b**) bubbly two-phase jet.

The representative images of each case, before and after the foundation cleansing test, are compared in Fig. 3. The images shown were converted to grayscale by applying the image-processing algorithm to the raw images (see Method). The general trends of the removal agree with those shown in Fig. 2. For both types of jets, the foundation in the central part was mostly removed in a circular shape, on which the jet flow was applied intensely. Meanwhile, the foundation separated from the core region tends to pile up on the surface and fall off in clumps, leaving a jagged border along the edge. With the single-phase liquid jet, the foundation remains in relatively thick clumps along the outer edge (Fig. 3a); however, the bubbly jet can remove the majority of the foundation, even at the edge region of the foundation, leaving only a thin layer (Fig. 3b). When the cleansing oil is used together with the single-phase liquid jet, it is now possible to remove the foundation on the edge and center area as effectively as using the bubbly jet without the aid of the chemical agent.

**Figure 4:**
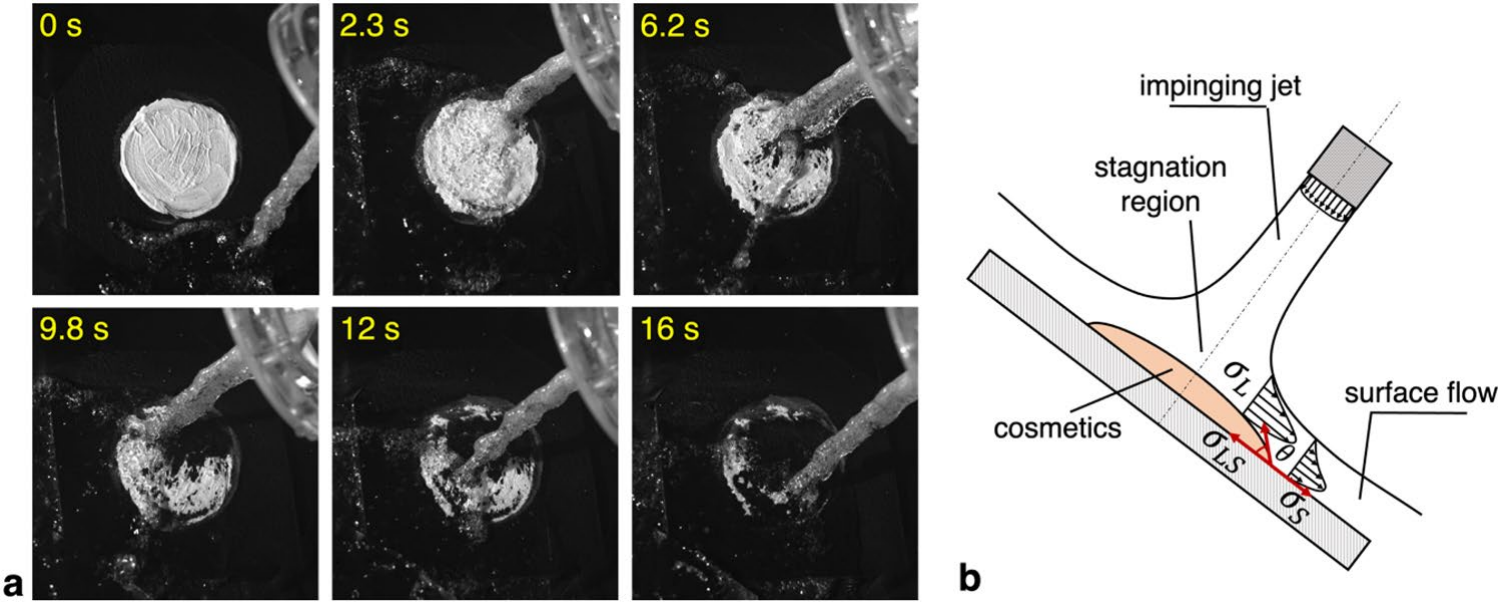
(**a**) Temporal variation of the foundation cleansing with the bubbly jet (water temperature of 40 °C). (**b**) Schematic diagram for the interaction between the impinging jet and cosmetics coated on the surface. Here, ${{\varvec{\sigma}}}_{{\varvec{L}}}$ and ${{\varvec{\sigma}}}_{{\varvec{S}}}$ are the surface free energy for liquid (cosmetics) and solid (synthetic leather), respectively, and ${{\varvec{\sigma}}}_{{\varvec{L}}{\varvec{S}}}$ is the interfacial energy between the liquid and solid.

For further understanding of the enhanced cosmetic removal with the bubbly jet, the sequential procedure of foundation cleansing for the case of a water temperature of 40 °C is given in Fig. 4a. At the initial stage of the cleansing process (2.3 s after the initiation), the outer edge of the foundation starts to peel off as the bubbly jet impacts on it, and the cosmetic layer becomes thinner. As the time elapses (after 6.2 s, for example), the cleaned area gradually expands along the radial direction. Later, the cosmetic layer where the bubbly jet initially contacts is completely removed from the surface (at 9.8 s), and the overall removal process is accelerated (for 10–16 s). The schematic diagram shown in Fig. 4b illustrates the cleaning mechanism of the impinging jet on the cosmetics adhered to the inclined surface, matching the setup of the present cleansing test (see Method). Before the jet flow is issued to the surface, the forces acting on the cosmetics lump (film) can be considered as the Young’s equation, which expresses the balance among surface energies involved, as expressed by ${\sigma }_{S}={\sigma }_{LS}+{\sigma }_{L}cos\theta$. Here, ${\sigma }_{L}$ and ${\sigma }_{S}$ denotes the surface free energy of the liquid (foundation) and solid (leather piece), respectively, ${\sigma }_{LS}$ is the solid–liquid interfacial energy, and $\theta$ is the contact angle. Following the analysis of van Oss et al.^33^, both of the two surface energies (${\sigma }_{L}$ and ${\sigma }_{S}$) can be divided into two components: the dispersive component (nonpolar Lifshitz-van der Waals) by the interatomic/molecular attraction and the polarity component (polar Lewis acid–base) from electrical attraction (Coulomb interaction) between permanent dipoles or between permanent dipoles and induced dipoles. ${\sigma }_{LS}$ incorporates dispersive and polarity components for both liquids and solids, allowing for the modification of interfacial energy and surface free energies in response to chemical reactions such as surfactant addition or temperature variation^34,35^. As the contact angle ($\theta$) approaches zero for the present film-like condition, the sum of ${\sigma }_{LS}$ and ${\sigma }_{L}$ balances with ${\sigma }_{S}$. As shown in Fig. 4b, the impinging jet flow on the foundation film spreads radially from the stagnation point, thereby forming a boundary-layer like surface flow^36^. As this surface flow pushes the foundation radially, the kinetic energy (${\sigma }_{FE}$) added to the surface free energy exceeds the sum of the cohesion energy of the foundation and the adhesion energy to the surface; ${\sigma }_{S}+{\sigma }_{FE}>{\sigma }_{LS}+{\sigma }_{L}$, where ${\sigma }_{FE}$ is determined by the mass flux and superficial velocity of liquid flow. With the bubbly jet, the increased superficial velocity of water (by bubble-induced drift) would lead to enhanced kinetic energy; however, with the single-phase jet, the foundation tends to be re-deposited on the edge because the kinetic energy is insufficient to detach the foundation film from the surface. With the addition of the surfactant (cleansing oil), on the other hand, the solid–liquid interfacial energy (${\sigma }_{LS}$) will be reduced so that the foundation can be removed even with the single-phase flow. In addition to increasing kinetic energy, the direct interaction between bubbles and foundation film would also accelerate the detachment of cosmetic film from the surface. That is, assisted by the inelastic microbubble-foundation collisions, the kinetic energy of the stationary foundation increases resulting in ${\sigma }_{S}+{\sigma }_{FE}+{\sigma }_{BE}\gg {\sigma }_{LS}+{\sigma }_{L}$, where ${\sigma }_{BE}$ denotes the kinetic energy gained by the foundation upon the impact with the bubbles. Even if the jet is fixed at a specific location (the fixed stagnation region noted in Fig. 4b) from the beginning of cleaning, the effect of bubbles causes the edges of other areas to softly deform (see Supplementary Movie S1). As seen in the movie, owing to the continuous collision with the bubbles, the foundation is removed almost entirely within 20 s by circling the jet impinging spot. This will be discussed in more detail later. Note that the soft circling of the jet around the cosmetic layer shown in the Supplementary Movie S1 was practically necessary to cover the discrepancy between the wider cosmetic layer (30–65 mm) compared to the jet width (12 mm). This slow circling motion of jet might cause an unsteady effect on the cleaning, but we think it will not change the proposed mechanism substantially considering its slower speed compared to the jet speed.

**Figure 5:**
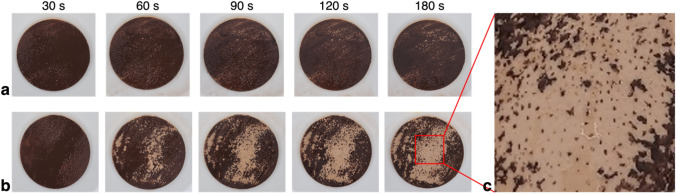
Removal of the foundation coated on the artificial cheek skin (water temperature of 25 °C): (**a**) single-phase liquid jet; (**b**) bubbly two-phase jet; (**c**) closed-up view of the pores on the skin surface.

The temporal variation of the cleansing patterns with and without the bubbles (water temperature of 25 °C) is also compared for the dark brown foundation on the artificial cheek skin. As shown in Fig. 5, the overall cleaning performance on the artificial skin was declined compared to that on the synthetic leather (Fig. 3), which is attributed to the deep pores distributed on the artificial skin and the stronger adhesion of the foundation on it. After 30 s of the cleaning, there is no discernible difference between the cases with and without the microbubbles, which can be visually detected. However, after 60 s have passed, the area where the foundation has been removed expands gradually by the bubbly jet (Fig. 5b), whereas the single-phase liquid jet still makes the foundation film slightly thinner and only exposes the skin surface sparsely (Fig. 5a). At around 180 s of the test, the removal of the foundation with the bubbly jet tends to be saturated, which is much better than the result achieved by the single-phase liquid jet. This is quite promising to indicate that the beneficial effects of bubbles in cosmetic cleaning will also be evident on the actual skin. On the other hand, the magnified view of the skin surface cleaned with the bubbly jet during 180 s shows that the remaining foundation mostly stays inside the pores, as opposed to the thoroughly cleansed smooth skin surface (Fig. 5c). Because the individual pore size is typically 250–500 μm^23^, the bubbles much smaller than the pore should exist in the bubbly jet to interact with the cosmetics inside the pores. Microbubbles of ***O***(10^1^–10^2^) μm used in this study are effective for the smooth surface of the skin but are not sufficient to thoroughly remove the cosmetics from skin pores; therefore, it seems favorable to introduce bubbles smaller than ***O***(10^0^) μm for such conditions. On the other hand, it will also be feasible by lowering the ${\sigma }_{LS}$ and ${\sigma }_{L}$ of the cosmetics while increasing the water temperature.

### Removal of the lip tint

**Figure 6:**
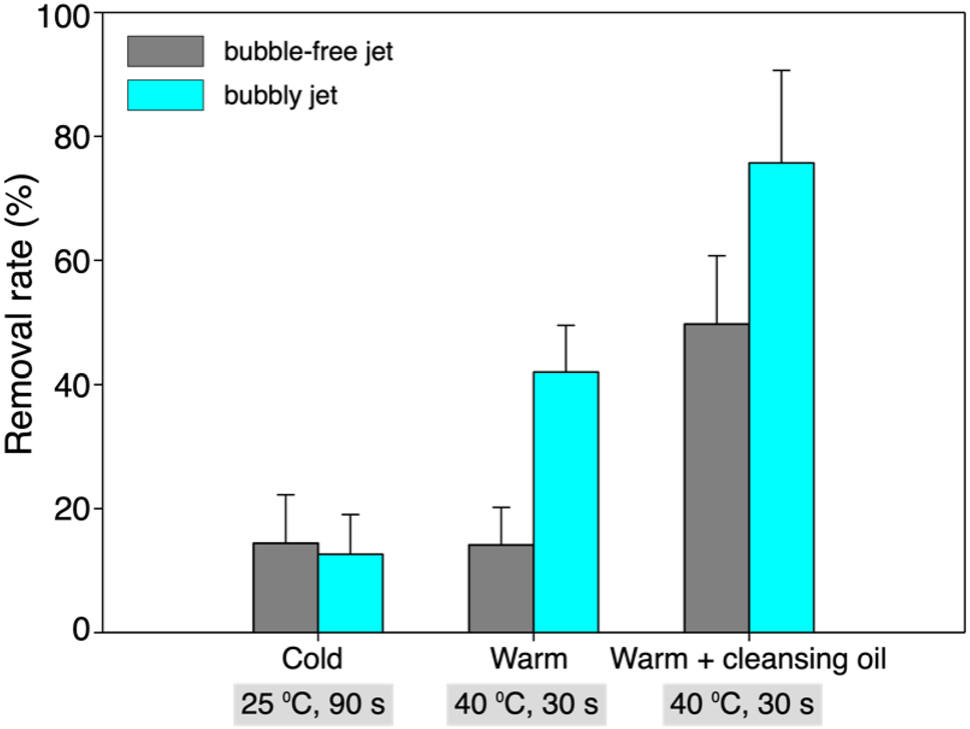
Removal rate of the lip-tint coated on the synthetic leather depending on the tested conditions (temperature of the water and duration of the application).

While the removal of the foundation on the synthetic leather and artificial skin was affirmative, here we discuss the removal of lip tint coated on the synthetic leather. Liquid-type foundation is a mixture of inorganic pigment powder with water, silicone, and oil emulsion. Lip tint is composed of a mixture of colorant and purified water, similar to lipstick without moisturizing ingredients such as oil or wax, providing much longer life time. Figure 6 compares the removal rate of the lip tint with and without the bubbles under the three different conditions that are the same as those considered for foundation removal. While the cleaning performance is enhanced with increasing water temperature and the addition of a chemical agent, similar to the case of foundation, the lip tint is in general more difficult to remove than the foundation, which agrees with our daily experience with other existing cleansing methods (note that lip and eye remover products are released on the market for this reason). Nonetheless, the enhanced cleansing with the bubbly jet is clearly observed. When the applied water temperature is 25 °C, the removal rates by both jets are comparable to each other, which are as low as 13–14%. As the water temperature rises to 40 °C and the cleansing oil is used together, on the other hand, the bubbly jet removes the lip tint as much as 76%, which is more than three times that at a lower water temperature; however, the removal rate of the single-phase liquid jet stays below 50%. It is noted that without the aid of cleansing oil, the performance of a single-phase liquid jet remains unaffected even by increasing the water temperature, which is different from the trends in removing the foundation. This indicates that the cleansing oil has little effect on the extremely high adhesion energy of lip tint, which can be overcome by the bubbly jet.

**Figure 7:**
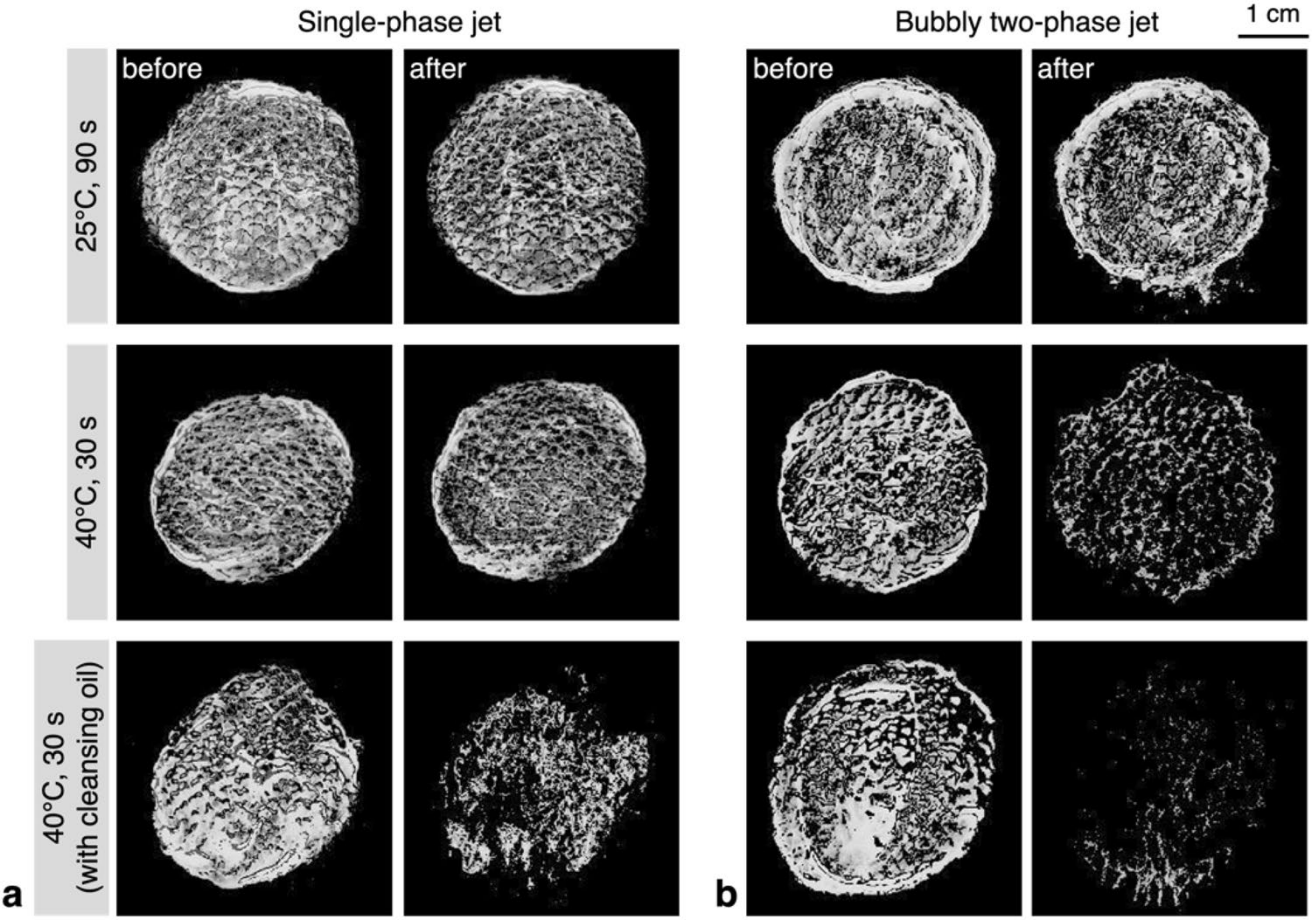
Results of the lip-tint cleansing test, of which the images were post-processed for quantification: (**a**) single-phase liquid jet; (**b**) bubbly two-phase jet.

In Fig. 7, we have compared the representative images obtained by the lip tint cleansing tests, which were post-processed for the removal rate to be quantified. As stated above, the effect of the bubbly jet is evident in the cases of higher water temperature. When the water temperature is relatively low, for both jets, the cosmetic surface layer is thinned down minimally and the tint is not washed away. With the warmer bubbly jet, the lip tint on the convex parts of the surface is mostly removed, but the residue remains along the concave grooves of the synthetic leather, similar to the pores on the artificial skin. The addition of the cleansing oil accelerates the bubble-assisted wash-away of the emulsified cleansing oil and cosmetics, resulting in a higher removal rate. Most of the residual cosmetic stays at the lower part of the circular pattern because gravity causes the cleansing oil to flow downward on the inclined test surface. However, with the single-phase liquid jet, due to the relatively low kinetic energy of the jet, the cleansing oil remaining on the cosmetic surface is first washed away, and then the emulsified cosmetics are removed sequentially, resulting in a lower removal rate.

### Further discussion on the role of bubbly jet

**Figure 8:**
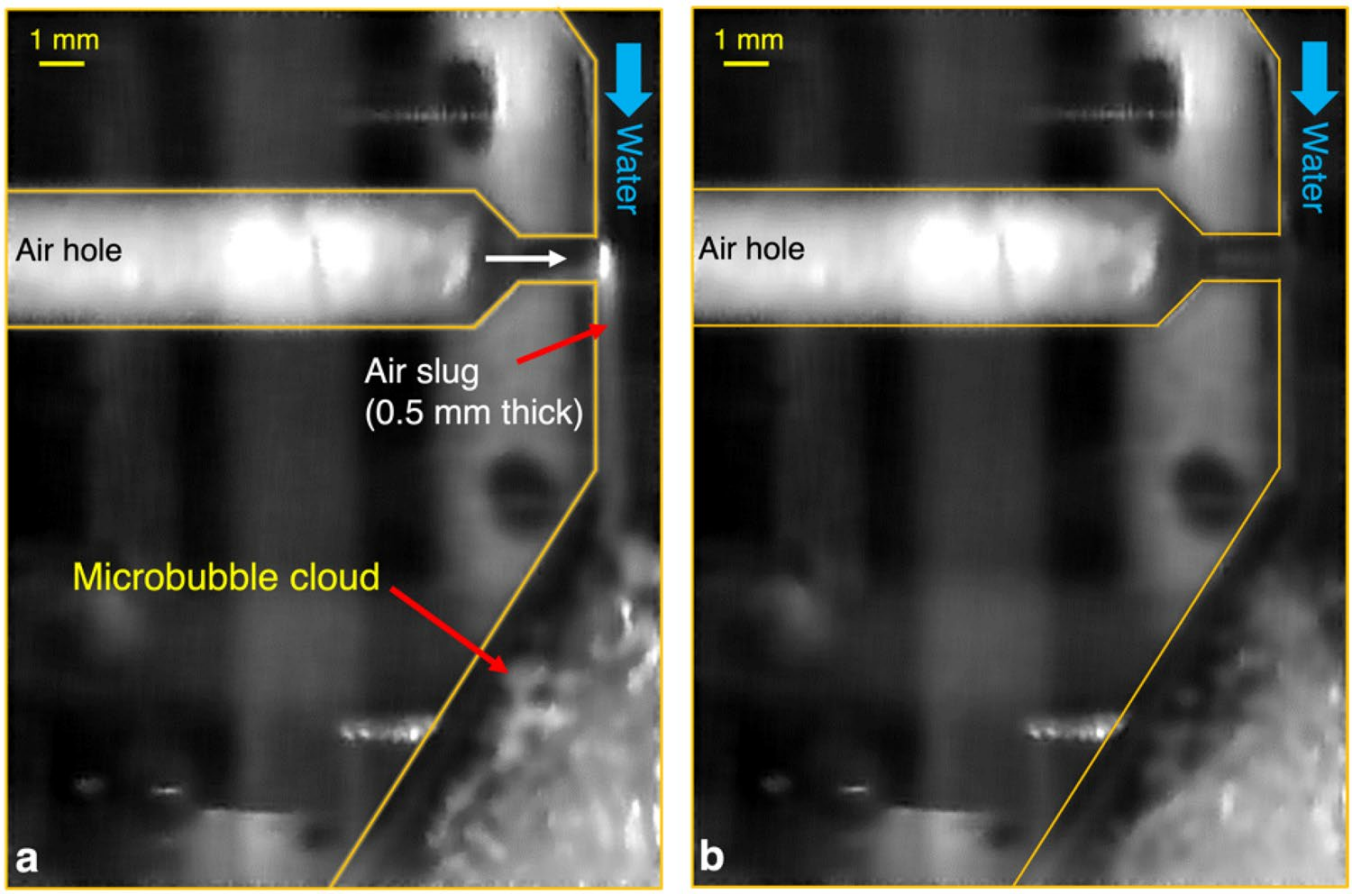
Enlarged images of the neck part in the bubble-generating nozzle: (**a**) open air hole; (**b**) closed air hole. Direction of the water flow inside the nozzle is from top to bottom, and the white color denotes the air.

As explained above, the micro-sized (***O***(10^1^—10^2^) μm) bubbles in the present bubbly jet have a direct (impact on the cosmetics) or indirect (increased superficial velocity of the water) effect on enhancing the efficiency of cosmetic cleansing. With the same volume flow rate of water applied, the water superficial velocity of the bubbly jet is dependent on the air volume fraction in the flow; that is, the water superficial velocity increases with increasing the air volume fraction for bubbly flows with micro-sized bubbles^32,37^. We visualized the flow inside the neck of the bubble-generating nozzle in order to measure the air volume fraction in the present bubbly jet (Fig. 8). The images were captured using a high-speed camera (SpeedSense M310, Dantec Dynamics) equipped with a 50-mm lens (Nikon) under a halogen lamp as illumination. The venturi nozzle draws the air slugs evolved from the entrained air through the air holes by the fast water flow at the neck, and the volume of the air slug increases with the water velocity^28^. In Fig. 8a, the typical size (thickness) of the air slug is calculated to be 0.5 mm; we consider that the air slug is shaped like a cylinder with a diameter of 0.5 mm. Assuming that the superficial velocities of water and air at the nozzle neck are equivalent, the air volume fraction ($2{D}_{slug}^{2}/{D}_{neck}^{2}$) is calculated to be 0.125 (see Table 1), where *D*_*slug*_ and *D*_*neck*_ denote the diameter of the air slug and nozzle neck, respectively. As noted, the water volume flux and jet diameter (measured at the nozzle outlet) of the single-phase and two-phase jets are identical. Since the air slugs occupy 12.5% of the cross-sectional area of the bubbly jet, the cross-sectional area occupied by water is smaller than that of the single-phase liquid jet. Thus, the superficial velocity of water becomes faster in the bubbly jet, increasing the kinetic energy of the surface (liquid) flow around the cosmetics film, which will additionally contribute to the enhanced cleansing performance of the two-phase bubbly jet.

**Figure 9:**
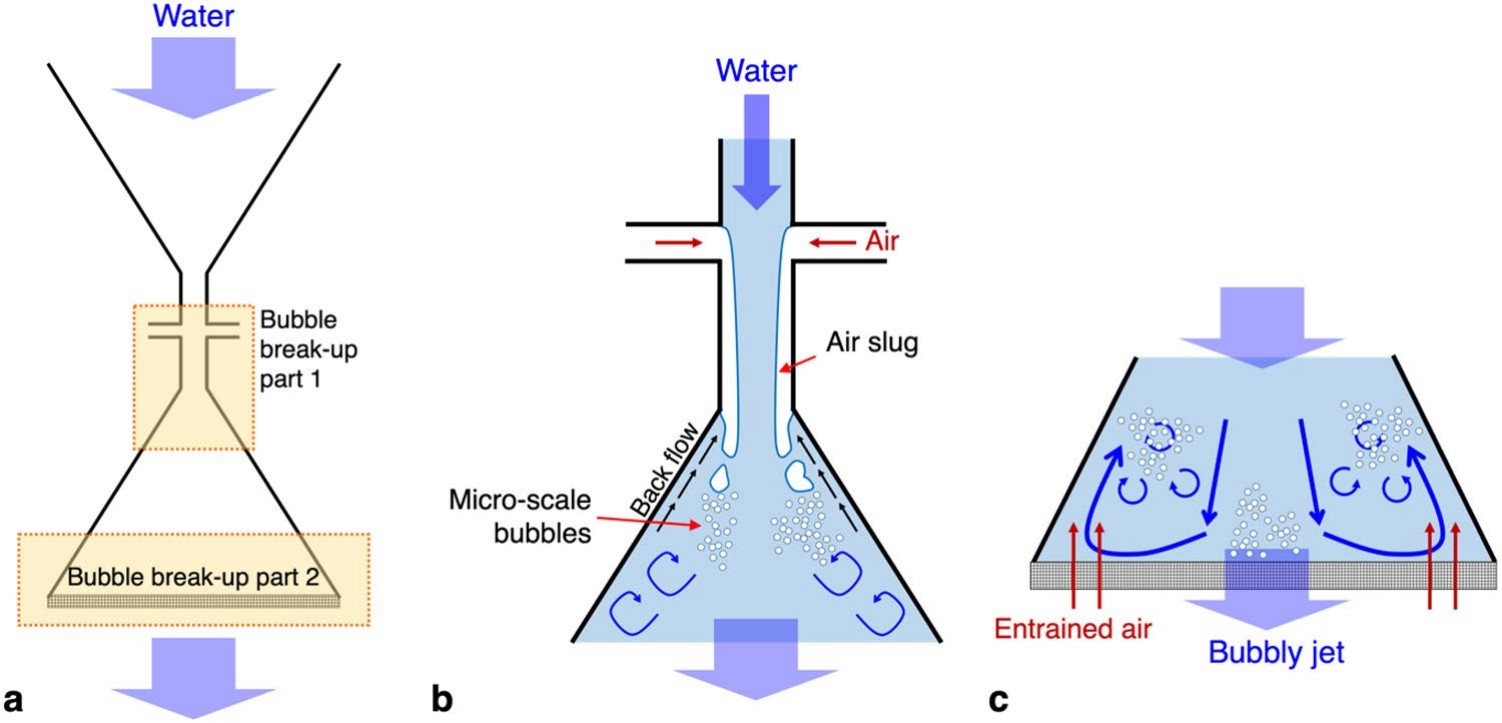
Schematics for the bubble generation mechanism in the present nozzle device: (**a**) locations where the bubbles are entrained and broken down; (**b**) bubble generation by the air slugs through the air hole; (**c**) bubble generation and segmentation by the entrained air through the mesh layers. Blue arrows denote the water flow direction.

Actually, the final air volume fraction in the bubbly jet is much larger than that we measured at the necking part. To understand this, the mechanism by which the bubbles are induced (entrained) in the nozzle is described in Fig. 9. For the present nozzle device, the bubbles are generated not only by the air slug entrained from the neck but also by the mesh installed at the nozzle outlet (Fig. 9a). As the cross-sectional area of the nozzle suddenly expands after the neck, the separated flow at the edge of the neck would form a wake region, in which the back flow in the opposite direction to the main stream is induced (Fig. 9b). As the air slug interacts with the re-circulating wake vortices, it would be dispersed into smaller bubbles. As shown in Fig. 8a, a cloud of microbubbles forms near the nozzle wall when the air holes are open; however, even if the air holes are intentionally blocked, it is still observed that the bubbles are induced along the expansion section of the nozzle path (Fig. 8b). For the present nozzle device, five dense mesh layers are overlapped at the nozzle outlet, and thus, the blockage ratio at the edge of the nozzle is extremely high, and the bubbly jet tends to be issued through the center area of the nozzle outlet. Therefore, it is understood that the air entrained through the mesh by the recirculating flow near the side wall of the nozzle breaks up into smaller bubbles by interacting with the swirling motions (Fig. 9c). In the present study, unfortunately, it was not allowed to measure the volume flux of the air entrained through the mesh directly, but it is clear that the air volume fraction in the bubbly jet was well over 12.5% based on our observations. Consequently, it is expected that the actual water superficial velocity of the bubbly jet will exceed the value shown in Table 1.

**Figure 10:**
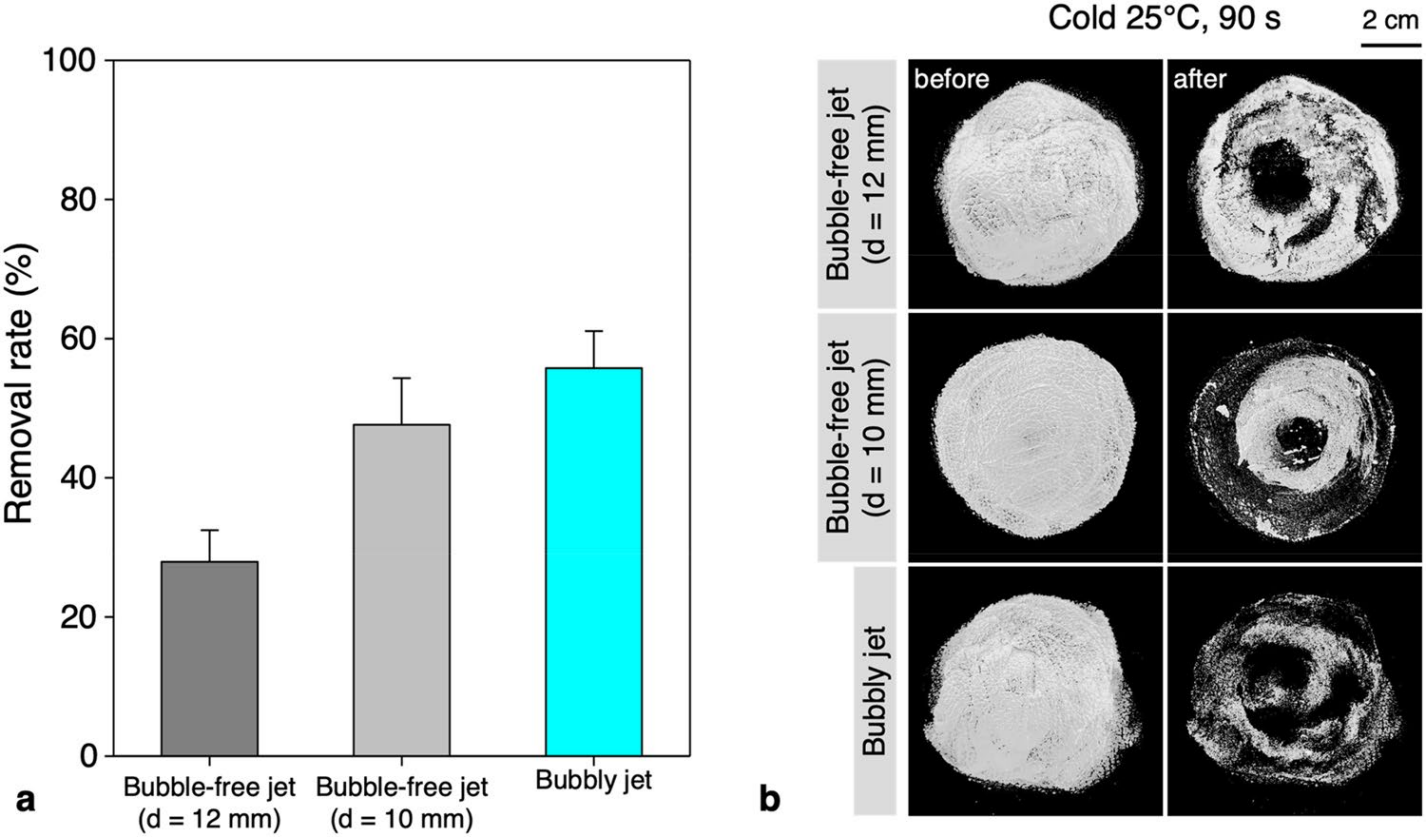
Effect of reducing cross-sectional area of the single-phase liquid jet in cold case: (**a**) removal rate of the foundation coated on the synthetic leather; (**b**) post-processed results of the foundation cleansing test. Here, d denotes the diameter of the water outlet.

While the increase in water superficial velocity caused by the reduction of effective cross-sectional area due to the existence of bubbles has a significant effect on increasing the cleaning efficiency, there might be additional effects by the bubbles. For example, the cosmetics are known to be aerophilic as they are mostly oil-based^38^, and thus the oil particles would tend to stick to the bubbles since they are intrinsically hydrophobic^29,39^. Then, the bursting of bubbles attached on the surface of the cosmetic layer will apply strong impulse at the instant of bubble rupture^21^, creating small cracks on it. The peel-off of the cosmetics can be initiated from these small cracks, further accelerating the cleaning process. To check the possibility of mechanism in addition to the enhanced inertia, we compared the removal rate of single-phase liquid jet with smaller cross-sectional area for the cold-water condition (Fig. 10). Here, the cross-section was reduced to 69.4% of the original value, smaller than the effective cross-sectional area of the bubbly jet (82.5% of the single-phase jet). With the enhanced inertia of water, the removal rate of the foundation (Fig. 10a) increases from 28 to 48%, however, it still did not reach the removal rate of the bubbly jet, and the discrepancy indicates that there exists additional removal mechanism by the bubbles. Representative images for each condition are shown in Fig. 10b. In the bubble-free jet (d = 10 mm) of reduced crossed-sectional area, larger area was cleaned off compared to the bubble-free jet with original outlet size (d = 12 mm), but the thin foundation layer still remained in the central part. In the case of the bubbly jet, the strong impulse generated by bubbles allows the two-phase jet to penetrate deeper into the cosmetic film, facilitating the more effective cleaning process.

## Concluding remarks

In the present study, we reported the development and demonstration of the effective removal of the cosmetics on the rough and porous surfaces using the two-phase bubbly jet flows even without the additional aids of the chemical cleansing agent. To achieve our goal, the hand-held nozzle device based on the venturi tube was designed to induce a bubbly jet with a targeted size of the bubbles of ***O***(10^1^–10^2^) μm, without the additional input of energy. That is, the air automatically entrained into the nozzle by the pressure difference evolves into bubbles, which will contribute to the enhanced kinetic energy of the flow by accelerating the effective liquid velocity. The cleansing performance of the micro-sized bubble-generating nozzle was tested on two types of cosmetics, foundation and lip tint coated on synthetic leather and artificial skin surfaces, varying the water temperature and adding the chemical detergent. Using the same volume flux of water, we confirmed that the bubbly two-phase jet shows much higher removal rates of foundation and lip tint compared to the single-phase liquid jet (without bubbles). The cosmetic cleansing by the bubbly jet is significantly enhanced, especially when the water temperature is higher, and it is also deduced that the bubbles can play the role of adding chemical agents. It is noted that the present bubbly jet with microbubbles only may not be sufficient to remove the cosmetics perfectly yet; however, it is quite promising to uncover that the two-phase jet with micro-sized bubbles is effective in removing the cosmetics coated with a strong adhesion force. That is, the bubble size and void fraction should be optimized depending on the applications, and it does not guarantee engineering success by simply introducing bubbly flows. The core mechanism of the bubble-assisted eco-friendly cosmetic cleansing method with less usage of water and chemical detergent, we are currently investigating to extend such a strategy to various cleaning applications such as semiconductor processing, electric home appliances, ship surface de-fouling, and so on.

The present study is a feasibility test to assess the viability of developing an eco-friendly cosmetic cleansing product. Our experimental findings have conclusively demonstrated that the presence of microbubble clusters significantly enhances the efficacy of cosmetics removal. Therefore, the results presented in this work are anticipated to serve as a meaning foundation for future endeavors. For the follow-up studies, we plan to optimize the geometry of the flow passage and rate of water flow to optimize bubbles in order to construct a prototype that aligns with the findings of the present investigation.

## Method

### Nozzle design for the bubbly jet

**Figure 11:**
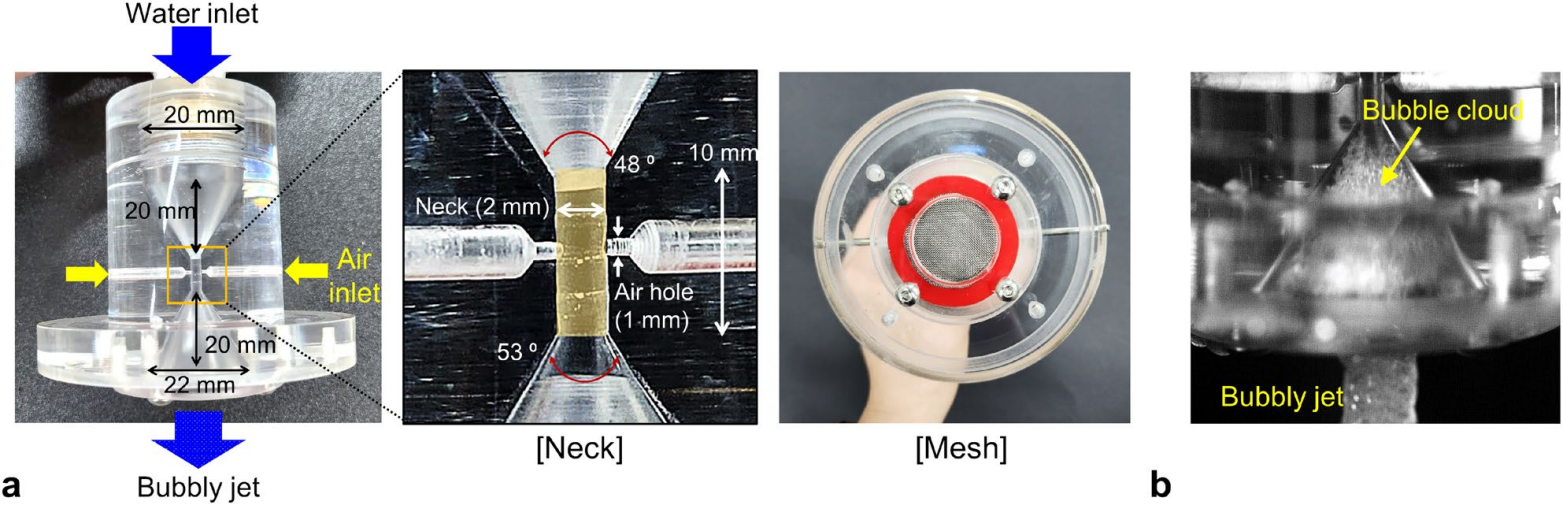
Bubble-generating venturi-type nozzle: (**a**) detailed design components; (**b**) bubble cloud visualization inside the nozzle.

There have been several types of microbubble generators (or aerators): ones in which the actively-injected gas phase is reduced to microbubbles by the high pressure or flow passage, and the others with which the ambient air is self-induced (entrained) passively into the bubbles^40^. Among the methods that does not require additional energy source to introduce gas flow, in the present study, we designed the acrylic venturi-type nozzle to generate microbubbles in a water jet without the additional input of external power (Fig. 11a). The present approach is characterized by the fast and substantial drop in pressure through the water passage, enabling the utilization of a standard tap water flow rate. The significant pressure drop can be induced by the sudden reduction in the cross-sectional area of the flow passage, realized by venturi shaping^28^, utilizing a ball^41^, or narrowing the passage area to a specific shape (adopted by many commercial aerators to induce nanobubbles). With the present design, on the other hand, the sudden pressure drop at the venturi neck where the ambient air is entrained passively, results in the formation of micro-bubbles. Based on its substantial microbubble output and simpler construction compared to conventional commercial aerators employing the same mechanism, we decided that the present design is more suitable for the removal of cosmetics (nanobubbles from the commercial aerators were reported to be more effective for water-soluble pollutant removal^24,25^). The working fluid is tap water that is filtered to eliminate the existing impurities, and the water flow passage inside the venturi nozzle gradually narrows from 20 to 2 mm. The length of the neck is 10 mm, after which the channel diameter recovers to 22 mm. That is, the contraction angle is 48 degrees and expansion angle is 53 degrees, which was determined based on the report that the bubble size is almost constant regardless of the flow rate when both the contraction and expansion angles are 60 degrees^42^.

On the side of the ‘neck’ part, two holes with a diameter of 1 mm are bored as a passage for air entrainment. Due to the change in cross-sectional area, the water flow is accelerated along the neck, resulting in a substantial pressure drop following the Bernoulli principle. Therefore, the ambient air can be continuously entrained into the nozzle through the large pressure difference at the neck, which further develops into the bubbles by interacting with the flow structures inside the nozzle^28^ (Fig. 11b). At the water outlet of the nozzle, five stainless mesh layers (wire diameter and aperture size are 0.1 mm and 0.3 mm, respectively) are mounted to contribute to the generation of the bubble cloud (Fig. 11a). A large portion of the water outlet area is effectively blocked by this mesh layer, causing a recirculating flow structure inside the expansion part of the nozzle. The locally reduced pressure by the recirculating flow would also draw air from outside the nozzle and increase the air fraction of the two-phase jet (detailed explanations are provided in the “Result” section).

### Measurement of bubble size in bubbly jet

To measure the distribution of bubble size, we performed the experiments in a compact water tank (0.3 × 0.3 × 0.3 m^3^) made of acrylic (see Fig. S1 in the Supplementary Materials). The present bubbly jet nozzle is located 1 cm above the free surface to ensure that the air–water interaction via the mesh installed at the nozzle inlet can be observed. Furthermore, it is challenging to measure the bubble size accurately if bubbles are too dense; thus, we captured the image of bubbles dispersing as soon as the bubbly jet is discharged from the nozzle and enters the water. To avoid interference from reflected light, the field of view (5.4 × 3.4 cm^2^) was positioned 1 cm below the water level (Fig. S1a). We used a high-speed camera (SpeedSense M310, Dantec Dynamics) to capture the images at 1000 frames per second with a resolution of 1280 × 800 pixels. As a light source, we used a Tungsten lamp (Openface 750W, ARRI) (Fig. S1b). The camera equipped with a 100-mm macro lens (Samyang) is positioned to face the frontal side of the tank, and subsequently, the lamp is located to illuminate the field of view from the side. The measurement was initiated 10 s after the entry of the bubbly jet into the water, and the water was simultaneously drained to maintain the same water level. After capturing the high-resolution images of bubbles, we conducted postprocessing to erase the background image and applied the binarization using the optimal threshold by Otsu’s algorithm^43^ (see Fig. S2 in the Supplementary Materials). Also, we eliminated the out-of-focus bubbles and noise existing in the images. Considering the jet impact velocity (~ 0.7 m/s) and distance to the free surface (= 10 mm) of the present setup, it is expected that the distortion of bubble size or additional bubble production due to the impact is insignificant^44^.

### Configuration of the cleansing experiments

**Figure 12:**
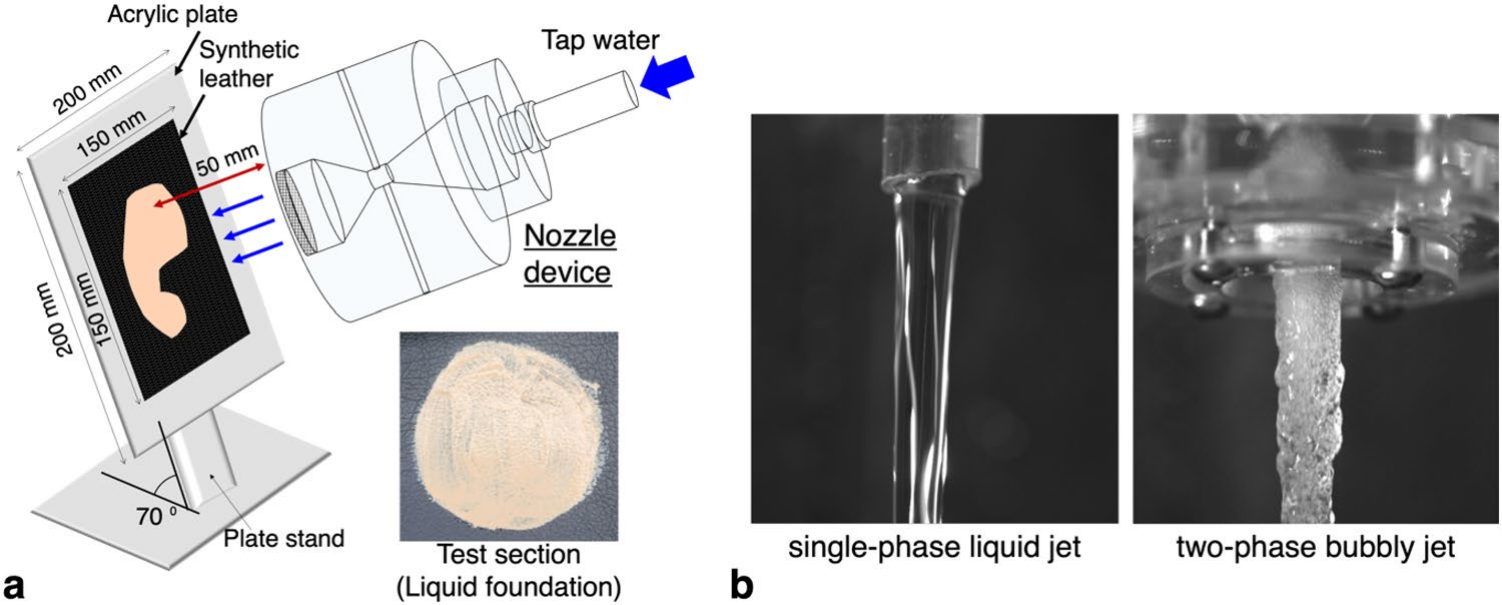
(**a**) Experimental configuration to test the cosmetic removal with the bubbly jet. (**b**) Images of the typical single-phase and two-phase jet flows.

To evaluate the cosmetic cleansing performance of the present bubbly jet, as a test surface, we consider the wrinkled synthetic leather (size of 100 × 100 mm^2^) (see Fig. S3 in the Supplementary Materials) attached to the acrylic plate (200 × 200 mm^2^). As shown in Fig. 12a, the cosmetics were applied on the surface and left to dry for two hours, and the jet (with and without bubbles) was applied while the cosmetic removal process was measured. The angle between the test section and the ground was fixed at 70 degrees to consider the angle of the user’s face when showering, and the position of the nozzle was adjusted so that the jet flow and the plate are perpendicular at a distance of 50 mm. In the present cleansing experiments, we considered the single-phase liquid jet together under the same condition as a reference case to evaluate the effect of bubbles (Fig. 12b). The single-phase liquid jet was ejected through a 16-mm diameter polyurethane tube (inner diameter: 12 mm). The volume flux of water was maintained to be constant at 4.5 lpm (liters per minute) for both single-phase and two-phase jets, which is similar to the volume flow rate from an ordinary tap water stand. As indicated in the inset of Fig. 12a, we evenly spread the specified amount of cosmetics in a circular pattern on the surface. The cosmetics used are two matte-textured liquid foundations (CLIO (light beige) and FENTY BEAUTY (dark brown)) and a thick-textured red lip tint (FORENCOS). Before applying the cosmetics, 0.3 g of moisturizing cream (for the foundation) and 0.05 g of lip balm (for the lip tint) were spread thinly and uniformly, respectively, on the surface to mimic the real makeup application procedure. On the surface with the moisture cream coated, 0.15 g of foundation was applied thinly in a 65-mm diameter circular pattern, and similarly, 0.05 g of lip tint was put in a circular pattern with a diameter of 30 mm on the surface with lip balm coated. In addition, the artificial cheek skin (BIOSKIN 10A, Beaulax Co., Ltd.) that replicates the human facial skin was used to assess the cleaning of liquid foundation that is known to be trapped in facial pores. The BIOSKIN has been used in various cosmetics-related studies. For example, Eudier et al.^45^ tested the ‘post-application stickiness’ of 10 cosmetic products and found little difference in stickiness between in vivo skin and artificial skin. Gore et al.^46^ observed that when 5 oil in water emulsions with different physical properties were applied to in vivo skin and BIOSKIN, the friction value and spreading rate (contact angle) were almost similar. Therefore, they concluded that it would be very helpful in future basic research such as evaluating the efficacy of new formulas on the skin. The same experimental protocol was applied to the tests with the artificial cheek skin.

In addition to the existence of bubbles, we also controlled the water temperature at 25 °C and 40 °C, and the time for the test was determined to be 90 and 30 s, respectively, considering that the cleaning performance in general becomes poorer with decreasing the water temperature. For the warmer water condition, the effect of additional (as small as 1 cc) cleansing oil was tested together. The removal experiment was conducted five times per condition. When the oil is rubbed on the cosmetics with a brush or fingertips, the frictional force may accelerate the substantial cosmetic emulsification. In this situation, it is impossible to precisely assess the cleaning effectiveness of the bubbly jet. Therefore, after dropping the cleansing oil, we piled another acrylic plate and a 2-kg weight on top of it and waited 1 min (for uniform oil distribution) (see Fig. S4 in the Supplementary Materials).

### Evaluation of the removal rate

**Figure 13:**
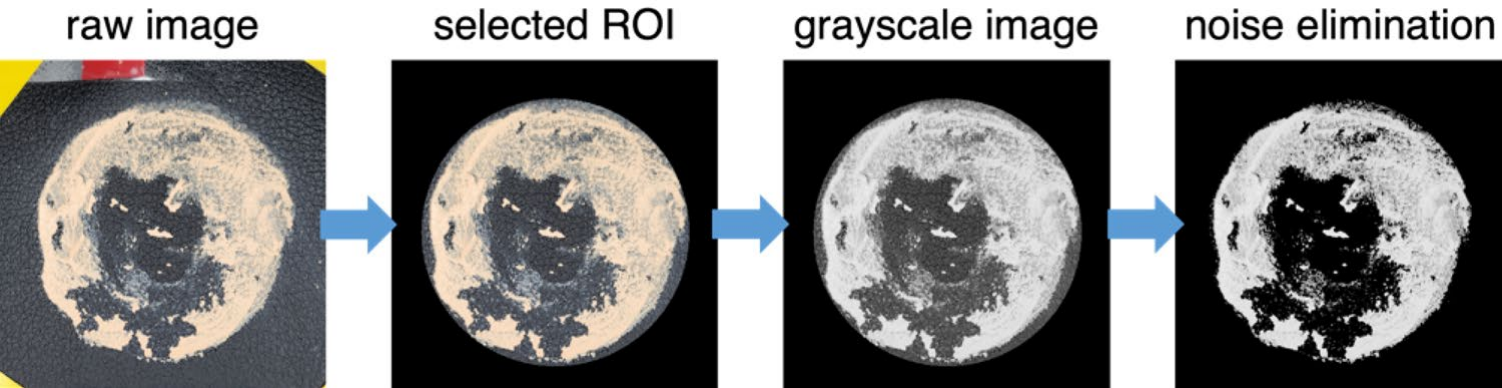
Image processing protocol to evaluate the removal rate of the cosmetics.

Before and after the cleaning tests, the images of cosmetics on the surface were taken using the camera embedded in the Samsung Galaxy Flip 4 under the LED ring light as a light source. The rate of cosmetic removal was determined by processing the raw images (Fig. 13). In the raw image, the region where the cosmetics were applied is first marked as the ROI (region of interest) and converted to the grayscale using the MATLAB in-house code. Simultaneously, all noises outside of the circular cosmetic zone are eliminated. After post-processing all images prior to and after the cleaning tests, the summation of the pixel intensity values (ranging from 0 (perfectly clean) to 1.0 (completely not cleaned)) is calculated for each image. Using these values, we calculate the cosmetic removal rate as 1.0–residual cosmetic fraction = 1.0–(intensity sum in ROI after cleansing)∕(intensity sum in ROI before cleansing).

### Supplementary Information

Supplementary Information.

Supplementary Figures.

Supplementary Video 1.

## Data Availability

All data generated or analyzed during this study are included in this published article and supplementary information files.
