# Supplementary material for: Toward the eco-friendly cosmetic cleansing assisted by the micro-bubbly jet
Source: Sci Rep. 2024 Apr 8;14:8189. doi: 10.1038/s41598-024-58968-x (PMC11001607; doi:10.1038/s41598-024-58968-x)
Supplement: Supplementary file 2 — Supplementary Figures. [file 41598_2024_58968_MOESM2_ESM.pdf]

Supplementary Materials for

Toward the eco-friendly cosmetic cleansing assisted by the micro-bubbly jet

Yeeun Kang[1,a)], Jooyeon Park[1,a)], and Hyungmin Park[1,2,b)]

[1]Department of Mechanical Engineering, Seoul National University, Seoul 08826, Korea.

[2]Institute of Advanced Machines and Design, Seoul National University, Seoul 08826, Korea.

[a)]Equally contributed to this work.

[b)]Author to whom correspondence should be addressed: hminpark@snu.ac.kr

Figure list

Figure S1. Experimental configuration to measure micro-sized bubble size in the bubbly jet. (a) Front view with a field of view. (b) Side view with the camera and light source.

Figure S2. Image postprocessing for determination of bubble distribution and size. (a) Raw image. (b) Lowering the brightness and removing the background. (c) Completed post-processing with binarization.

Figure S3. Detailed images of the synthetic leather pieces and artificial skin: (a) leather for the foundation; (b) leather for the lip tint; (c) artificial skin.

Figure S4. The method of uniformly applying cleansing oil to the test section without any direct contact.

Movie list

Movie S1. Temporal variation of the liquid foundation film cleansing with the warm (40°C) bubbly jet

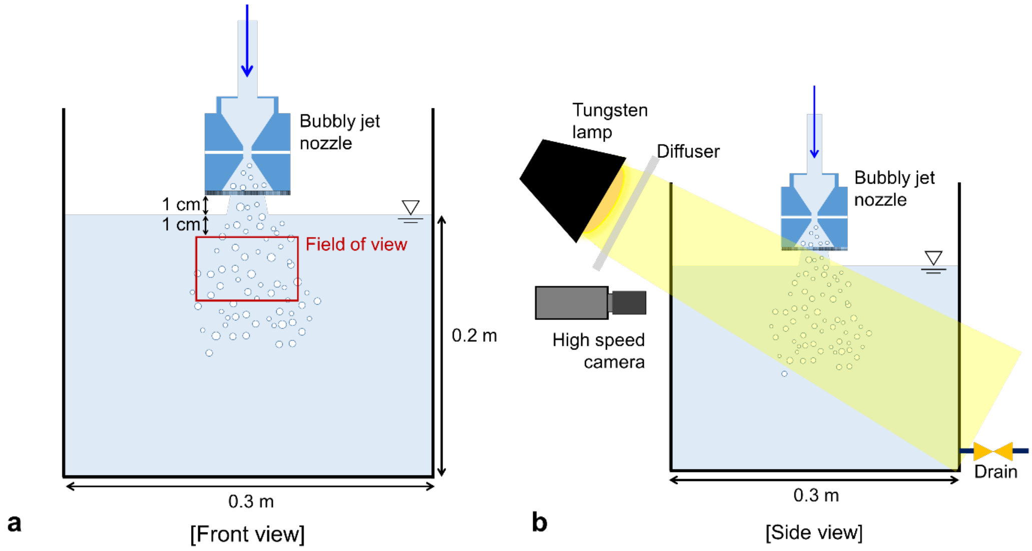

Figure S1. Experimental configuration to measure micro-sized bubble size in the bubbly jet. (a) Front view with a field of view. (b) Side view with the camera and light source.

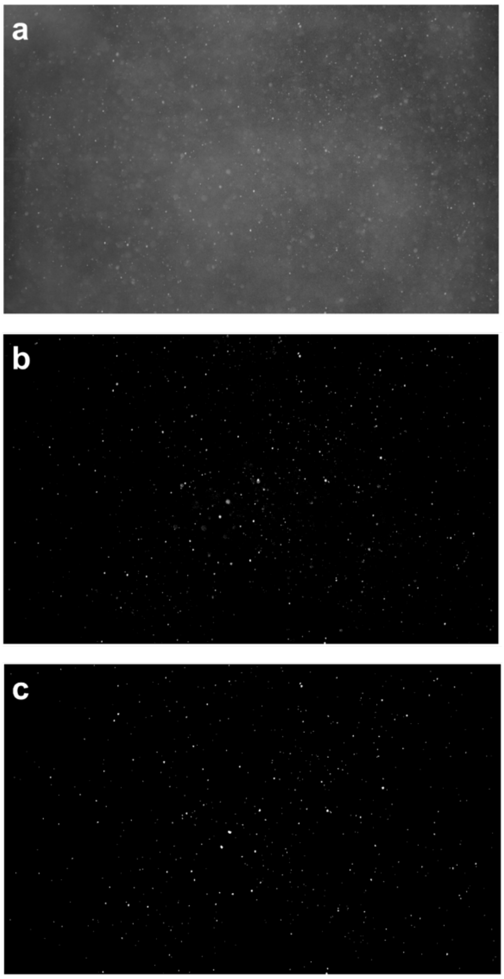

Figure S2. Image postprocessing for determination of bubble distribution and size. (a) Raw image. (b) Lowering the brightness and removing the background. (c) Completed post-processing with binarization.

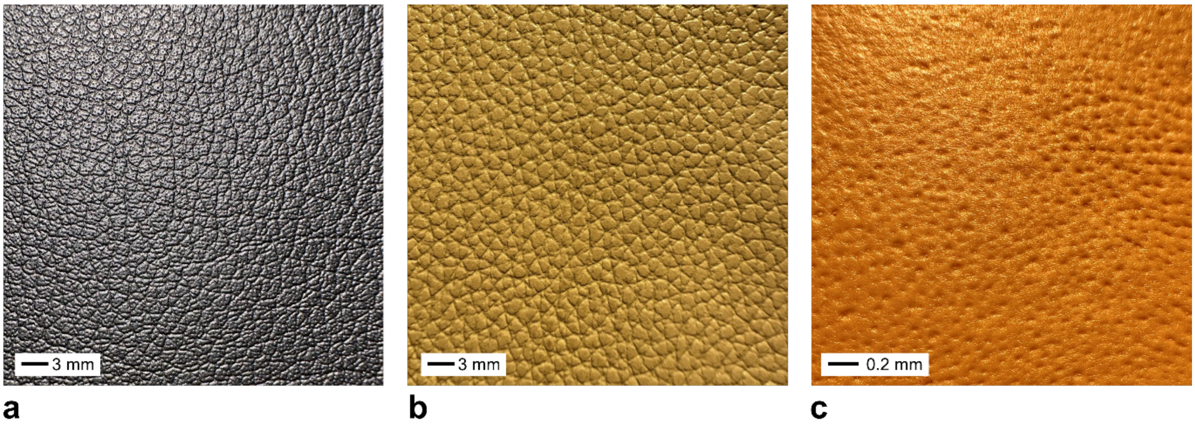

Figure S3. Detailed images of the synthetic leather pieces and artificial skin: (a) leather for the foundation; (b) leather for the lip tint; (c) artificial skin.

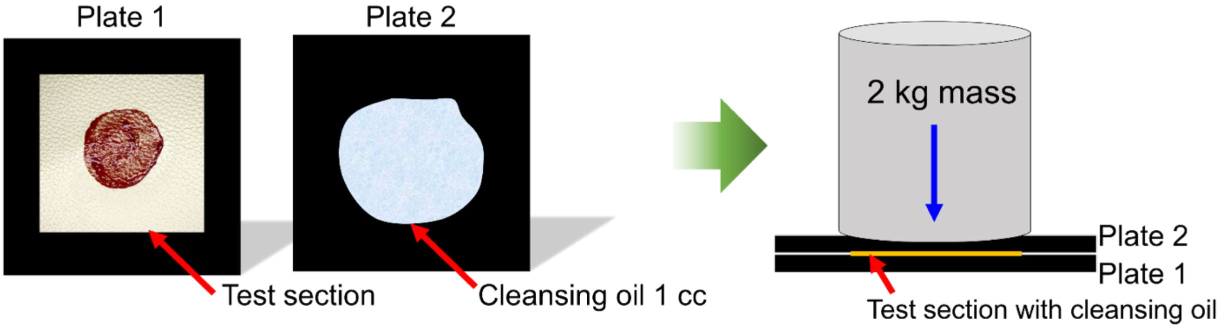

Figure S4. The method of uniformly applying cleansing oil to the test section without any direct contact.
